# The memory of thin polymer films generated by spin coating

**DOI:** 10.1140/epje/s10189-022-00205-2

**Published:** 2022-05-25

**Authors:** Günter Reiter, Farzad Ramezani, Jörg Baschnagel

**Affiliations:** 1 Institute of Physics, Albert-Ludwigs-Universität Freiburg, 79104 Freiburg, Germany; 2 Institut Charles Sadron, Université de Strasbourg and CNRS, 67034 Strasbourg Cedex, France

## Abstract

We present results from isothermal and temperature-sweep creep experiments adapted to filaments which were derived from spin coated and subsequently crumpled thin polystyrene films. Due to the existence of residual stresses induced by preparation, the filaments showed significant shrinkage which we followed as a function of time at various temperatures. In addition, the influence of preparation conditions and subsequent annealing of supported thin polymer films on shrinkage and relaxation behavior was investigated. The temporal evolution of shrinkage revealed a sequence of relaxation regimes. We explored the temperature dependence of this relaxation and compared our observations with published results on drawn melt-spun fibers. This comparison revealed intriguing similarities between both systems prepared along different pathways. For instance, the magnitudes of shrinkage of melt-spun fibers and of filaments from crumpled spin coated polymer films are similar. Thus, our results suggest the existence of generic mechanisms of “forgetting”, i.e., how non-equilibrated polymers lose their memory of past processing events.

**Graphical abstract:**

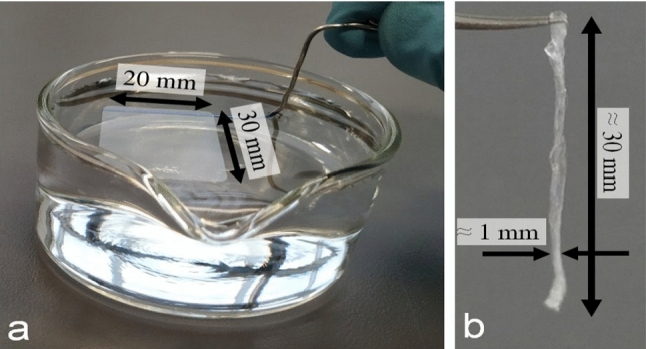

## Introduction

In processing polymers, various pathways can induce significant stretching and alignment of polymer chains, in particular when strong flow fields, fast cooling or steps of rapid solvent evaporation are involved [1–6]. Processing-induced deviations in the statistics of chain conformations from an equilibrium distribution are accompanied by a reduction in conformational entropy [7–10]. Non-equilibrated polymers reflect experiences of the past and thus may be considered as their “memory”, often expressed through extraordinary and unexpected properties [4, 5, 11]. For example, melt-spun and drawn fibers or so-called shape memory polymers remember their processing conditions and often consist of highly deformed and oriented polymer chains, which allow for changes in shape and/or significant contraction of their length [12–20]. It has been proposed that non-equilibrated polymers can overcome local potential barriers via appropriate thermal “activation” of frozen-in entropic forces and contraction can start even in the glassy state [6, 13–17]. Furthermore, isothermal shrinkage of stretched and oriented polystyrene in melt-spun and drawn polymer fibers was shown to follow an Arrhenius-type temperature dependence with an activation energy around 100 kJ/mol [13, 15], even at temperatures below the glass transition [13, 17, 18]. Keeping the length of a fiber constant and increasing temperature at a constant rate, the generation of transient stresses can be measured [17, 18], revealing the complex spectrum of relaxation regimes of stretched and oriented structural elements in polymer fibers. For example, measured stress-temperature curves were interpreted with a model representing a sequence of three relaxation regimes acting at distinctly different characteristic timescales [18].

In fibers, frozen-in non-equilibrated polymers (or segments of them) can contract upon thermal activation [6, 17]. The corresponding relaxation processes also influence surrounding polymers. In the vicinity of relaxing non-equilibrated polymers, compressive stresses (often termed “thermal stresses”) may appear [13–18]. When no external force (load) is applied, these thermal stresses can lead to significant shrinkage. Relaxations at a segmental level allow to transform (part of) the gain in conformational entropy into mechanical energy, which can also be used for lifting attached loads [19]. In the present study, partially inspired by observations made for melt-spun and drawn polymer fibers, we explore if related relaxation processes occur in spin coated thin polymer films [21].

For the preparation of thin polymer films on various types of substrates, one often starts from polymer solutions. Preparation pathways may involve significant and rapid changes in concentration and molecular mobility. For example, thin polymer films can be generated by spin coating, a frequently employed pathway for their preparation. There, fast evaporation of solvent represents the key step, inducing a rapid transition from separated polymers in a rather dilute solution to a dry and glassy film of (partially) interpenetrating polymers. As a consequence of rapid solvent evaporation, the time available for polymer relaxations is often much shorter than the time required for complete equilibration [22]. Thus, as shown in various studies [21–29], polymers in very thin spin coated films are never completely equilibrated. In glassy films, they adopt frozen-in, anisotropic, non-equilibrated chain conformations, which, in turn, generate forces acting on the polymer chains (termed “residual stresses”). The presence of residual stresses in spin coated polymer films has been demonstrated and quantified through various phenomena, e.g., the formation of dewetting holes or micro-cracks [22, 24–29], the deflection of micro-cantilevers [30], the contraction of films on liquid surfaces [31] or via an expedited onset of wrinkling instabilities [32]. There, the magnitude of residual stresses has been reported to vary in a wide range from a few kPa to hundred MPa.

Explored by dewetting experiments, a systematic dependence of properties of thin polymer films on the degree of deviation from equilibrium controlled by varying preparation pathways has been established [22]. For example, a power law relation between the amount of residual stresses and a preparation parameter $\wp$ was deduced, defined as the ratio of the time required over the time allowed for equilibration, i.e., the ratio of the relaxation time of the polymer fluid over the time available for the polymers to relax during the evaporation step of the spin coating process. However, in dewetting experiments like the ones described in [22], the influence of the substrate and the geometry of the employed dewetting process impede an unambiguous determination of the amount of residual stresses and the corresponding relaxations. Thus, as a complementary approach, we searched for a possibility to characterize out-of-equilibrium properties of thin polymer films which does not require substrates and is based on a simple geometry. Our recently developed creep experiment was adapted to measure changes in length of polymer filaments derived from spin coated and subsequently crumpled thin films [21]. In this previous study, we explored the impact of non-equilibrated polymer chains in these films by illustrating the film relaxation process via the lifting of macroscopic loads (${\sigma }_{\mathrm{load}}$). The lifting of the loads implies that there is an oppositely oriented effective force counteracting the weight force, the origin of which we associate with preparation‐induced residual stresses (${\sigma }_{\mathrm{res}}$). Based on this approach, we visualized the force corresponding to ${\sigma }_{\mathrm{res}}$ on a macroscopically observable scale by determining strain recovery in thin spin coated polymer films via the contraction of a filament at a fixed temperature and as a function of the applied load. In the present study, we put a particular focus on the temporal evolution of residual stresses in these thin polymer films at different temperatures. In addition, we compare published experimental results on thermal stresses observed in drawn melt-spun fibers presented in the literature [12–19] with residual stresses in the here studied filaments derived from spin coated thin films. Furthermore, we present results on how the amount of recovered strain depends on sample preparation and on prior annealing of the initially supported films.

## Experimental section

The present study is based on the experimental procedures and results described in our previous publication [21], where experimental aspects, including sample preparation, spin-coating procedure, effects of annealing, surface tension, etc. have been discussed in detail. Here we only repeat a few salient features essential for the current study and refer the interested reader to Ref. [21] for further information.

### Preparation of crumpled films (filaments) from spin coated films

For the present study, we used polystyrene with weight-average molar mass of 524 or 925 kg/mol and a dispersity *Đ* = 1.03, supplied by Polymer Standards Service GmbH, Mainz, Germany. All polystyrene films were prepared by spin coating, which proceeds in a sequence of stages [33–35]: First, a polymer solution is deposited on a rotating substrate. Due to the centrifugal forces, the solution flows in the radial direction and an excess is ejected off the edge of the substrate. Subsequently, in the remaining film of polymer solution, centripetal forces are balanced by capillary forces because the sharp edge of the substrate can be represented as an "effective contact angle" which results in retaining the fluid from flowing over the edge. At this stage, the film formation process is dominated by solvent evaporation. During the evaporation stage, polymers progressively adsorb onto the substrate. For long chains, this absorption may be strong [4, 36]. Consequently, chains are essentially pinned on the substrate and therefore can only change their conformations in the direction perpendicular to the substrate, resulting in tensile residual stresses in the plane of the film [37, 38]. Thus, as the lateral dimensions of this “solution film” are not changing (i.e., the film is not shrinking), only the thickness is decreasing during solvent evaporation [22]. The polymer concentration increases during the evaporation stage of spin coating [33–35, 38] and polymer chains interpenetrate progressively more [22]. This process is accompanied by a decrease in polymer mobility and an increase of the glass transition temperature of the polymer solution [22, 27, 28, 38]. Upon evaporation, the polymer solution approaches its glass transition, polymers freeze and cannot equilibrate their conformations anymore [22]. For polystyrene solutions, this happens at a solvent volume fraction of 10–30% [38]. Thus, chain interpenetration and equilibration are stopped before all solvent is evaporated [37, 38]. Accordingly, we expect that non-equilibrated polymer chains in the resulting dry film (after evaporation of the residual solvent) experience forces which either push or pull on their segments.

**Fig. 1:**
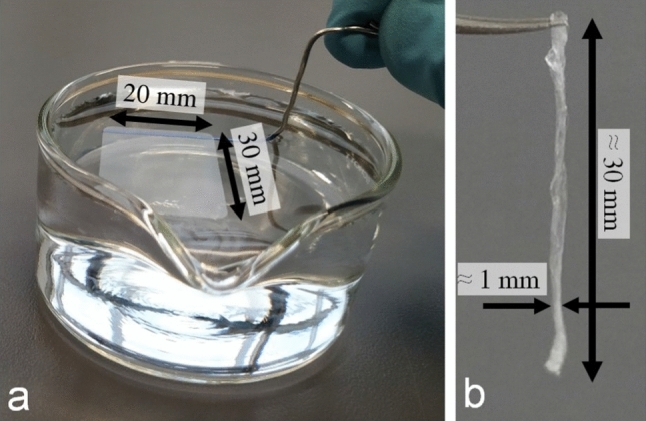
Photographs of the key stage for the preparation of polymer filaments from spin coated thin films. **a** The spin coated polymer film of defined size is floating on a water surface. At the top end of the floating film, the metal wire has been already introduced from below. **b** When lifting the film off the water surface, it crumpled and resulted in a filament

For our study, we transformed spin coated films into filament-like samples, a procedure introduced in a previous study [21]. Here, we only give a brief description. We first floated a freshly spin coated film (to be referred to as “as-cast film” in the remainder of the text) of given lateral dimensions from a solid substrate (i.e., silicon wafer and also mica) onto a clean water surface. Then, we picked this floating film at one side with a thin metal wire and slowly lifted it off the water surface. During lifting, the film deformed and crumpled laterally, resulting in a fiber-like object, which we called “filament”. Photographs of the key stage of filament preparation are shown in Fig. 1. We defined the cross-sectional area $A$ of the filament from the initial dimensions of the floated film, i.e., from the product of thickness and width of the film. To assure the removal of residual solvent, all samples were dried at a temperature well below the glass transition, i.e., at room temperature for at least 12 h. During this drying step, no significant physical ageing occurred (see the Supplemental Material of [21]), i.e., non-equilibrated polymers remained frozen-in.

### Adapted creep experiment for crumpled spin coated films

For the determination of molecular relaxations within thin polymer films and the corresponding changes of rheological properties in time, we designed for our filaments a method similar to the set-up used for macroscopic creep experiments, i.e., we determined the rheological response under the action of an applied stress [39, 40]. First, we attached at each end of the filament solid holders prepared from aluminum foils which were folded and tightly squeezed around the filament. One end of the filament was fixed to a stand while the other end was allowed to move freely under the influence of an applied load ${\sigma }_{\mathrm{load}}$ and in response to the relaxation of residual stresses ${\sigma }_{\mathrm{res}}$ acting on the polymers in the filament. In the here presented experiments, the load $\sigma_{{{\text{load}}}} = mg/A$, with $m$ being the attached mass, varying between 1 and 5 mg, and $g$ the gravitational acceleration, *g* = 9.8 ms^−2^, was kept at a small value and varied between about 4 kPa and 10 kPa by attaching aluminum foils of different size and weight. Small loads were applied to prevent the filament from bending and folding back on itself which would hamper an accurate length measurement. For ${\sigma }_{\mathrm{load}}<10\,\mathrm{kPa}$, our previous experiments [21] showed that the filaments did not show significant re-elongation at long times, i.e., such small loads did not have a significant impact on the shrinkage process.

We note that typically the mass of the freely hanging polystyrene filament was around 10–50 µg and can therefore be neglected with respect to the attached mass $m$. The whole set-up (stand with the hanging filament) was placed rapidly (within ca. 10 s) inside an oven at atmospheric pressure (Heraeus vacuum oven VT 6025, Thermo Electron LED GmbH, Germany) equipped with a double Pyrex glass door. Before introducing the sample, the oven was preheated to a desired temperature. The time when the door of the oven was closed was defined as the starting time ($t = 0$) of our experiment.

Changes in length $L\left(t\right)$ of the filament as a function of time $t$ were monitored with a camera placed outside the oven. $L\left(t\right)$ was deduced from the taken images using ImageJ software [41]. From the measurement of $L(t)$, we deduced the time dependence of the Hencky strain $\varepsilon \left( t \right)$ according to $\varepsilon \left( t \right) = \ln \left[ {L\left( t \right)/L\left( 0 \right)} \right]$, with *L*(0) being its initial length at *t* = 0. With this approach, we could determine $\varepsilon \left( t \right)$ with an uncertainty of ca. $\pm 0.003$ at a time resolution of ca. $\pm 10\,{\text{s}}$.

Since the typical cross-sectional area of the filament is of the order of $A \sim 10^{ - 9}\, {\text{m}}^{2}$ and the thermal diffusivity $D_{{\text{T}}}$ of polystyrene is of the order of $D_{{\text{T}}} \sim 10^{ - 7}\, {\text{m}}^{2}\,{\text{s}}^{ - 1}$ for the studied temperature range [42], the typical diffusion time $t_{{{\text{Diff}}}}$ of temperature across the sample is $t_{{{\text{Diff}}}} \sim 10^{ - 2}\, {\text{s}}$, which is much shorter than our time resolution (of the order of seconds). Thus, we can assume isothermal conditions to hold for our creep experiments.

Since the crumpled film exhibited a substantial amount of free interface that might tend to disappear upon heating to the working temperature of the experiment, it cannot be excluded a priori that stresses due to surface tension might affect our results. Thus, in our prior study [21], we have addressed this point by a control experiment where we removed (almost) all residual stresses by annealing the film on a liquid surface for 30 h at 130 °C, prepared a filament by crumpling the film and performed a creep experiment. The behavior of such an annealed crumpled film showed only negligible contraction, demonstrating that surface tension effects did not result in contraction of annealed filaments.

A typical response of a polymer melt to an applied stress consists of a rapid elastic elongation accompanied by viscous flow. In our previous study [21], we have demonstrated that filaments made from sufficiently annealed films showed indeed such a behavior. By contrast, as-cast films contracted with time, exhibiting a negative creep behavior. It has been suggested that shrinkage of polymer fibers can be interpreted as a measure of internal stresses [6, 12–21].

## Results and discussions

### Isothermal creep experiments at varying temperatures

In order to investigate the temperature $\left( T \right)$ dependence of the relaxation processes of preparation-induced residual stresses that caused macroscopic contraction, we performed creep experiments adapted to filaments [21] made from crumpled 200 nm polystyrene ($M_{{\text{W}}} = 925\, {\text{kg}}/{\text{mol}}$) films. We note that for this film thickness no deviations of the glass transition temperature from its bulk value ($T_{{\text{g}}} \approx 100\, ^\circ {\text{C}}$) have been observed (see e.g., [4]).

**Fig. 2:**
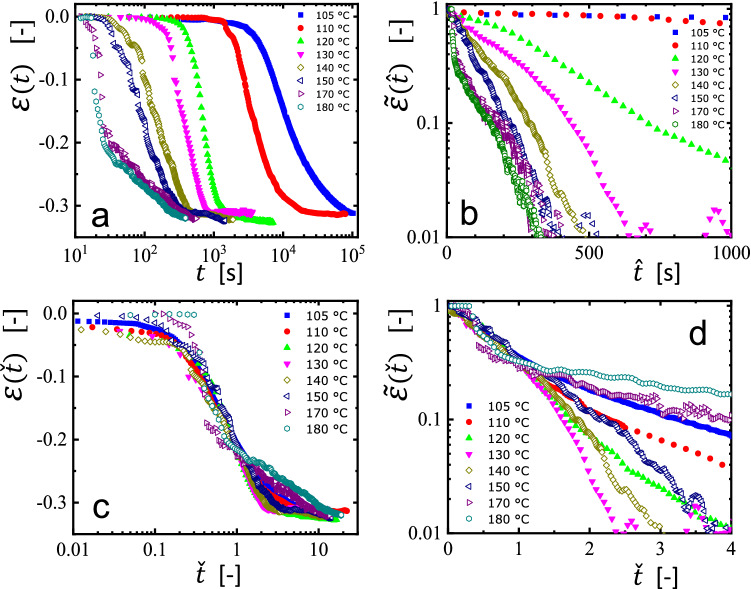
Influence of temperature on the strain response of non-equilibrated polystyrene filaments: **a** Linear-logarithmic representation of the strain response $\varepsilon \left( t \right)$ at an applied load of 6 kPa measured at different temperatures for filaments made from 200 nm films of polystyrene (*M*_W_ = 925 kg/mol), spin coated at 2000 rpm. **b** Same data as in (**a**), represented in a logarithmic-linear form of $\tilde{\varepsilon }\left( {\hat{t}} \right) = \left[ {\varepsilon_{{{\text{limit}}}} - \varepsilon \left( {\hat{t}} \right)} \right]/\varepsilon_{{{\text{limit}}}}$ as a function of $\hat{t} = \left[ {\left( {t - t_{0} \left( T \right)} \right)} \right]$. **c** Same data as in (**a**), represented in a linear-logarithmic form of $\tilde{\varepsilon }\left(  \check{t} \right)$ as a function of $\check{t} = \hat{t}/\tau \left( T \right)$. **d** Same data as in (**a**), represented in a logarithmic-linear form of $\tilde{\varepsilon }\left( \check{t} \right)$ as a function of $\check{t}$. See text for further details

In Fig. 2a, we present our results as a function of time for the change of the Hencky strain $\varepsilon \left( t \right)$ of the filaments, on semi-logarithmic scales, at an applied load of around 6 kPa, measured at different temperatures. In order to analyze these curves, we followed an approach [15] proposed for melt-spun and drawn polymer fibers. There, shrinkage started only after a temperature-dependent induction time $t_{{{\text{induct}}}} \left( T \right)$ which (roughly) followed an Arrhenius-type representation, yielding for many polymers an activation energy of the order of ca. 100 kJ/mol. Interestingly, not only $t_{{{\text{induct}}}} \left( T \right)$, but also the shrinkage process itself (characterized by the decay time $\tau_{{{\text{fiber}}}} \left( T \right)$) followed an Arrhenius-type representation, yielding a similar activation energy.

According to the proposed model [13–17], shrinkage of oriented amorphous polymers in these fibers is initially hampered due to constraints between neighboring molecular segments. In [15], $t_{{{\text{induct}}}} \left( T \right)$ was interpreted as the time required for initiating the shrinkage process through rearrangements facilitated by internal stresses within a certain volume, representing a cluster of cooperatively acting molecules or segments. Internal stresses effectively reduced the activation barrier for rearrangements within the corresponding activation volume. A “four-state model” was proposed [13–17] which successfully described experimental observations of the temperature dependence of $t_{{{\text{induct}}}} \left( T \right)$ and $\tau_{{{\text{fiber}}}} \left( T \right)$.

Inspired by the analysis of the shrinkage of fibers [13–17], we adopted this approach for the contraction of filaments of crumpled spin coated films, yielding in essence the results shown in Fig. 2b–d. Correspondingly, in analogy to $t_{{{\text{induct}}}} \left( T \right)$ defined in [15], we deduced from the traces shown in Fig. 2a within the resolution of our experiments an induction time $t_{0} \left( T \right)$ as the time when the length of the filament decreased by 1% of its initial value. The scatter of the data points shown in Fig. 2a introduced an uncertainty represented by error bars. In addition, we also determined the limiting value $\varepsilon_{{{\text{limit}}}}$, the lowest value of $\varepsilon \left( t \right)$ measured at the longest times. To obtain the values of $\varepsilon_{{{\text{limit}}}}$, we averaged the values of the last 10–20 data points. The scatter of these data points is reflected by error bars. We note that reaching $\varepsilon_{{{\text{limit}}}}$ does not necessarily imply that all polymers in the filament were equilibrated. Interestingly, independently of the temperature chosen for the creep experiment, all filaments showed the same maximum contraction, i.e., the values of $\left| {\varepsilon_{{{\text{limit}}}} } \right|$ did not differ significantly for experiments performed at various temperatures. As all filaments were prepared in an identical way, we anticipated equal values of the residual stress $\sigma_{{{\text{res}}}}$ in each filament. Accordingly, as $\left| {\varepsilon_{{{\text{limit}}}} } \right|$ did not depend on the temperature at which contraction occurred, we assumed that this value is approximately equal to the steady state recoverable strain $\varepsilon_{{\text{r}}}^{0}$ as a consequence of $\sigma_{{{\text{res}}}}$ for which we deduced a value of the order of 100 kPa [21].

In Fig. 2b, the relative changes in length of the filaments, represented by $\tilde{\varepsilon }\left( {\hat{t}} \right) = \left[ {\varepsilon_{{{\text{limit}}}} - \varepsilon \left( {\hat{t}} \right)} \right]/\varepsilon_{{{\text{limit}}}}$, are traced versus shifted times, defined as $\hat{t} = \left[ {\left( {t - t_{0} \left( T \right)} \right)} \right]$ for all curves shown in Fig. 2a. Using semi-logarithmic scales, the initial stages of contraction of all curves can be approximated by straight lines, suggesting that the initial contraction of $\tilde{\varepsilon }\left( {\hat{t}} \right)$ followed an exponential function. Thus, we assumed that a significant part of the contraction of the filaments can be characterized by a single temperature-dependent relaxation time $\tau_{1} \left( T \right)$. From Fig. 2b, we deduced the values of $\tau_{1} \left( T \right)$ as the time when the value of $\tilde{\varepsilon }\left( {\hat{t}} \right) = \frac{1}{{\text{e}}} \cong 0.37$ was reached.

Intriguingly, while the decay of $\tilde{\varepsilon }\left( {\hat{t}} \right)$ initially followed an exponential function characterized by $\tau_{1} \left( T \right)$, deviations from such a simple behavior became clearly evident when tracing either $\varepsilon \left( \check{t} \right)$ (see Fig. 2c) or $\tilde{\varepsilon }\left( \check{t} \right)$ (see Fig. 2d) as a function of normalized time $\check{t}$, defined as $\check{t} = \hat{t}/\tau \left( T \right)$. The representation in Fig. 2c highlights the region where the length of the filament remained initially almost unchanged (induction period), the region where significant shrinkage was detected (contraction process) and the region where the length did not change much further (related to $\varepsilon_{{{\text{limit}}}}$). Interestingly, Fig. 2d strongly suggests the existence of a sequence of several relaxation regimes, as indicated by a splaying of the curves for values of $\tilde{\varepsilon }\left( \check{t} \right) < 0.3$, i.e., a change in the temperature-dependent decay rate. Within the examined range, only the data taken at $T = 120 ^\circ {\text{C}}$ showed a single slope, indicating a single relaxation time. However, at all other temperatures either a slower or a faster decay was observed for $\tilde{\varepsilon }\left( \check{t} \right) < 0.3$. At long times, for values of $\check{t} > 2$, all curves indicated a constant slope, suggesting a second stage of exponential decay. Thus, in addition to the two characteristic times already deduced form previous experiments on shrinkage of drawn melt-spun fibers, we introduced the second stage of shrinkage (characterized by the second decay time), which, however, only became detectable once the first stage reduced the length of the filament significantly. Our experimental data (see Fig. 2d) suggested that this second stage set in once the relative length has decayed to $\tilde{\varepsilon }\left( \check{t} \right) < 0.3$. This value was reached approximately after the characteristic decay time $\tau_{1} \left( T \right).$ Accordingly, assuming that the curves of Fig. 2d represent a sequence of only two exponential processes, we have extracted a second relaxation time $\tau_{2} \left( T \right)$ from the decay for values of $\tilde{\varepsilon }\left( \check{t} \right) < 0.3$. For obtaining an estimate of $\tau_{2} \left( T \right)$, we have subtracted the contribution with the decay time $\tau_{1} \left( T \right)$ from $\tilde{\varepsilon}\left( \check{t} \right)$ and determined the time when the remaining strain decayed to 0.37 of its value at $\check{t} = 0$.

**Fig. 3:**
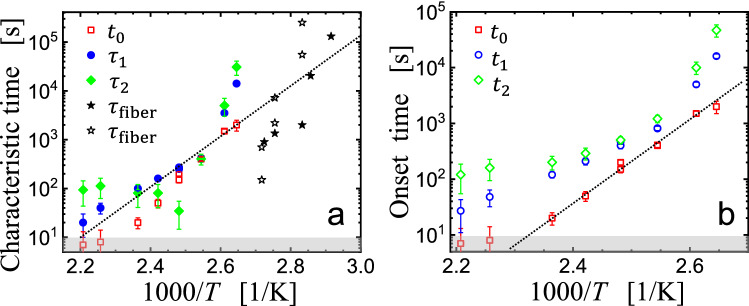
Arrhenius-type representation of the various times deduced from the analysis of the data shown in Fig. 2. **a** $t_{0} \left( T \right)$ (squares), $\tau_{1} \left( T \right)$ (circles) and $\tau_{2} \left( T \right)$ (lozenges). Values of $\tau_{{{\text{fiber}}}} \left( T \right)$ which were deduced from Fig. 9 of reference [19] and from Figs. 2 and 3 of reference [12] are represented by full and open stars, respectively. **b** $t_{0} \left( T \right)$ (squares), $t_{1} \left( T \right) = t_{0} \left( T \right) + \tau_{1} \left( T \right))$ (circles) and $t_{2} \left( T \right) = t_{1} \left( T \right) + \tau_{2} \left( T \right)$ (lozenges). The gray-shaded regions in (a) and (b) indicate that, for our experimental approach, characteristic times less than ca. 10 s are prone to significant uncertainties. For guidance, the dotted lines in **a** and **b** indicate an activation energy with a value of 80 kJ/mol and 150 kJ/mol, respectively

The values of the various times [$t_{0} \left( T \right)$, $\tau_{1} \left( T \right)$ and $\tau_{2} \left( T \right)$] extracted from the analysis of the curves of Fig. 2 are shown in the Arrhenius-type representation of Fig. 3. In addition, we have added values $\tau_{{{\text{fiber}}}} \left( T \right)$ which we extracted from references [10, 17] where the contraction of melt-spun polystyrene fibers has been investigated. The values of $\tau_{{{\text{fiber}}}} \left( T \right)$ were extracted in the same way as applied for determining the values of $\tau_{1} \left( T \right)$ from Fig. 2.

In order to distinguish the differences between incubation time and decay time, we have introduced onset times for the various stages. For the first stage of filament contraction, the onset time is given as the induction time $t_{0} \left( T \right).$ Supported by the data shown in Fig. 2d, we assumed that the second stage started after a duration $\tau_{1} \left( T \right)$ of the first stage, yielding the onset time $t_{1} \left( T \right)$ for the second stage: $t_{1} \left( T \right) = t_{0} \left( T \right) + \tau_{1} \left( T \right)$. Following similar assumptions, we defined $t_{2} \left( T \right) = t_{1} \left( T \right) + \tau_{2} \left( T \right)$. These results are shown in Fig. 3b.

We emphasize that in Fig. 3 we compared samples derived along strongly different preparation pathways. The fibers in references [12, 19] were spun under strong elongational flow from a polystyrene melt and had a diameter of some 100 µm. In addition, the fibers were drawn during the spinning process, introducing significant stretching and alignment of the polymers. As verified by birefringence measurements, the fibers consisted of highly oriented polystyrene molecules [19]. By contrast, our filaments were prepared from spin coated films of 200 nm without any stretching during the process of filament formation [21].

While the Arrhenius-type representation of Fig. 3 cannot be considered as a proof that the same mechanisms are at work in shrinking fibers and filaments, it is interesting to note that we could identify similar stages in the shrinkage process (incubation time, decay time, temperature dependence …). By this comparison, we do not imply that mechanisms of shrinkage for fibers and filaments were identical in all aspects. However, given the differences in preparation pathways and experimental approaches, it is quite surprising that we observed for the filaments of crumpled spin coated polymer films an almost equal degree of shrinkage [19, 21]. For fibers, it was shown that $t_{{{\text{induct}}}} \left( T \right)$ had similar values as $\tau_{{{\text{fiber}}}} \left( T \right)$ and both showed a similar temperature dependence (activation energy)[15]. As can be seen from Fig. 3, the here deduced values of $t_{0} \left( T \right)$ and $\tau_{1} \left( T \right)$ and the values of $\tau_{{{\text{fiber}}}} \left( T \right)$ [12, 19] cover a similar but not overlapping temperature region in an Arrhenius diagram. It is therefore tempting to link both data sets by an Arrhenius fit, yielding an activation energy of the order of 100 kJ/mol.

We admit that such a crude analysis may be debatable. However, in order to be able to compare published results from the shrinkage of fibers with our measured contraction of filaments, we believe that the use of an analogous analysis approach is justified. Clearly, in order to close the gap with the results for fibers, it would be desirable to improve the statistics of our film data and to extend our experiments to lower temperatures. Such an extension should reveal whether or not the characteristic times of the films continuously crossover to the times found for fibers. Inspection of Fig. 3 suggests that the resulting temperature dependence of the characteristic times would likely obey a stronger than Arrhenius increase with decreasing temperature, that is, would display an activation energy that increases upon cooling.

For fibers of oriented polymers, it has been proposed [16] that frozen-in entropic forces are sufficient to overcome local energy barriers even below the glass transition temperature, i.e., these forces are strong enough to allow for contraction also in the glassy state. The temporal evolution of shrinkage forces and their changes upon heating at a constant rate have be found to depend on the magnitude of internal stresses stored during fiber formation [16].

The here observed values of the activation energy of the order of 100 kJ/mol are reasonably comparable with values derived from the temperature dependence of relaxation times $\tau_{{{\text{res}},{\text{dew}}}}$ deduced from dewetting experiments of polystyrene films on a solid substrate [22, 24–29] or on a liquid glycerol surface [31]. As summarized in [29] in a comparison of results from various types of measurements, the temperature dependence of all these relaxation times is characterized by an activation energy between ca. 30 and 110 kJ/mol. It has been proposed that segmental rearrangements were at work which allowed to relax stresses in non-equilibrated polymer films [29].

### Isothermal creep experiments for films prepared under different spin coating conditions

**Fig. 4:**
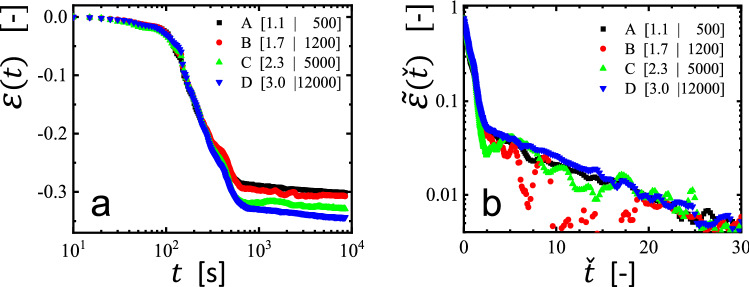
The macroscopic behavior of filaments made from crumpled spin coated films depends on preparation conditions. **a** Linear-logarithmic representation of the strain response $\varepsilon \left( t \right)$ of filaments made from 105 nm thick polystyrene (${M}_{{\text{W}}} = 524{\text{ kg}}/{\text{mol}}$) films, measured at 130 °C with an applied load of 5 kPa. The filaments coded as [$c|v$] in the figure were derived from films obtained by spin coating of polymer solution of concentration *c* (in [% w/w]) at a rotational speed *v* (in [rpm]). **b** The same data represented as $\tilde{\varepsilon}\left( \check{t} \right)$, using $t_{0} \left( T \right) = 150\, {\text{s}}$ and $\tau_{1} \left( T \right) = 250\, {\text{s}}$ for all curves. The deduced values of $\left| {\varepsilon_{{{\text{limit}}}} } \right|$ differed between these four curves and ranged from 0.302, 0.307, 0.330, to 0.344 for curves *A* $\to$ *D*, respectively

Recent dewetting experiments on spin-coated thin polymer films have demonstrated that the behavior of polymer films is not governed by film thickness only [22, 29]. Films of a given thickness but prepared along widely varying pathways yielded significant differences in $\tau_{{{\text{res}},{\text{dew}}}}$. An appropriately defined preparation parameter $\wp$ revealed quantitative correlations between preparation pathways and the macroscopic behavior of polymer films. $\wp$ is essentially proportional to the ratio of the time $\tau_{{{\text{relax}}}}$ required over the time $\Delta t$ allowed for equilibration. $\Delta t$ is proportional to the time of solvent evaporation from the spin coated solution film. Chain relaxation is expected to be low or even negligible for $\tau_{\text{relax}} > \Delta t$. Inspired by these results deduced from dewetting experiments, we explored if such an influence of preparation pathways can also be detected by the here employed creep experiments on filaments made from spin coated films. Thus, we have prepared filaments from equally thick polystyrene films spin coated at different spinning rates $\nu$, measured in rounds per minute –${\text{rpm}}$, from solutions with an appropriately adjusted polymer concentration $c$. In contrast to the films employed for the experiments shown in Fig. 2, we used thinner (105 nm) films of a lower molecular weight (${M}_{{\text{W}}} = 524\,{\text{kg}}/{\text{mol}}$). The measurements were carried out at $130\, ^\circ {\text{C}}$ with an applied load of 5 kPa. The resulting curves are shown in Fig. 4a. We note that all curves of $\varepsilon \left( t \right)$ superposed quite well at short times but differed distinctly at later times, demonstrating that $\varepsilon_{{{\text{limit}}}}$ depends significantly on conditions of film preparation.

Filaments derived from films prepared at higher spinning rates, i.e., for large values of $\wp$ [22], showed higher values of $\left| {\varepsilon_{{{\text{limit}}}} } \right|$. From curves *A* ⟶ *D* of Fig. 4a, we deduced values of $\left| {\varepsilon_{{{\text{limit}}}} } \right|$ ranging from 0.302, 0.307, 0.330, to 0.344, respectively. Interestingly, in the representation of $\tilde{\varepsilon }\left( \check{t} \right)$ shown in Fig. 4b, all curves showed the same behavior. Independently of the preparation conditions, we obtained $t_{0} = 150\, {\text{s}}$, $\tau_{1} = 250\,{\text{s}}$ and $\tau_{2} \approx 2700\,{\text{s}}$ for all curves. Only the value of $\left| {\varepsilon_{{{\text{limit}}}} } \right|$ increased when the time available for equilibration of polymer conformations became shorter, represented by an increase in the preparation parameter defined in [22]. For the data shown, the corresponding preparation parameters varied roughly by a factor of 4.

The fact that the values of the characteristic times did not depend on the preparation pathway may hint at relaxation mechanism(s) which are not affected by the conditions of spin coating, even for varying amounts of residual stresses. Possibly, in line with previous results on drawn melt-spun polymer fibers [13–17], the mechanism of relaxing residual stresses is related to processes at a segmental level (we like to call it an “untightening of knots”). One may speculate that these processes are independent of the acting stresses which, in turn, are proportional to the number density of the required segmental relaxation steps within the sample, similar to the “four-state model” proposed in references [13, 15]. However, further studies are required for gaining profound insight into the underlying mechanism(s).

### Behavior of films prepared under different spin coating conditions

One of the main advantages provided by the here employed creep experiment is the possibility to characterize relaxation processes in thin polymer films without any possible perturbations due to interactions with a substrate. At the same time, a comparison of the creep behavior of filaments derived from as cast films with the one of filaments made from annealed films may provide information about the role of interactions with a solid substrate on relaxation processes of residual stresses. Thus, we have performed two-stage experiments: First, polymer films supported on mica were annealed for various times at a set temperature. Subsequently, these annealed films were transformed to filaments for measuring their contraction in a creep experiment performed at 120 °C with an applied load of 6 kPa.

**Fig. 5:**
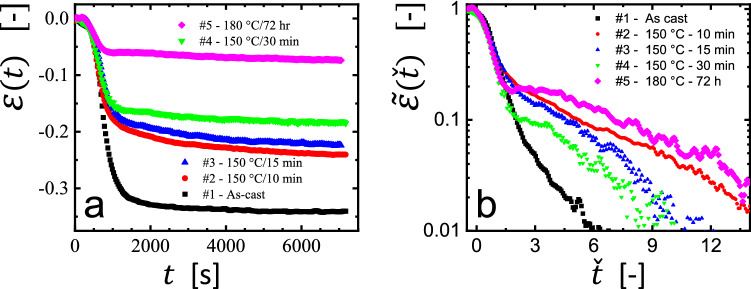
Relaxation of residual stresses in supported films requires long times in comparison to freely hanging filaments. **a** The strain response $\varepsilon \left( t \right)$ of polystyrene ($M_{{\text{W}}}$ = 925 kg/mol) filaments made from films annealed on mica at different conditions, as noted in the figure. These results are presented in comparison with $\varepsilon \left( t \right)$ measured for an as-cast film. The thickness of all films before and after annealing was around 200 nm. The creep experiments were carried out at 120 °C with an applied load of 6 kPa. **b** The same data represented as $\tilde{\varepsilon }\left( \check{t} \right)$, using $t_{0} \left( T \right) = \tau_{1} \left( T \right) = 410\,{\text{s}}$ for all curves. The deduced values of $\left| {\varepsilon_{{{\text{limit}}}} } \right|$ differed between these four curves and ranged from 0.344, 0.242, 0.185, to 0.073 for the curves #1 $\to$ #5, respectively

Figure 5 shows the corresponding results of the creep experiments on filaments made from 200 nm thick polystyrene (Mw = 925 kg/mol) films spin coated and then annealed on mica at 150 °C for different times of 10, 15 and 30 min. For comparison, we also show in the same figure results for a filament made from an as-cast film with the full initial residual stresses and for a filament made from a film annealed for 72 h at 180 °C for which we expected to observe no residual stresses because the longest equilibrium relaxation time (reputation time) is of the order of 100 s.

We observed that the degree of contraction decreased as the annealing conditions became more severe, reflecting relaxations of residual stresses during annealing on the solid substrate. From the as cast films studied in Fig. 2, we observed that in freely hanging crumpled films (filaments) residual stresses relaxed rapidly, with a value of $\tau_{1} = 100\, {\text{s}}$ for the measurement performed at $T = 150\, ^\circ {\text{C}}$. However, as can be seen in Fig. 5, even after annealing the film on mica at $T = 150\, ^\circ {\text{C}}$ for 30 min, the corresponding filament still contracted by about 18% in length, i.e., only less than 50% of the total residual stresses could be relaxed by annealing on the substrate. This suggests that relaxations of residual stresses and thus molecular mobility is retarded when the film is supported by a solid substrate, consistent with previous observations [43–46]. Even when the film was annealed at the higher temperature of 180 °C for the much longer time of 72 h, we still could measure a value of $\left| {\varepsilon_{{{\text{limit}}}} } \right| = 0.073$. In this context, we would like to mention that Thomas and Steiner [30] have observed also a sequence of two relaxation processes (sum of two exponential functions) for the decay of residual stresses at 155 °C in a 100 nm thick spin coated polystyrene film, yielding long characteristic times of 0.9 and 30 h. We conclude that a complete removal of preparation-induced residual stress in supported thin polymer films requires extremely long annealing times. These results may imply that for adsorbed polymers different or additional relaxation mechanisms are at work, which proceed much more slowly.

From the representation of $\tilde{\varepsilon }\left( \check{t} \right)$ shown in Fig. 5b, we observed differences in the decay of $\tilde{\varepsilon }\left( \check{t} \right)$, especially at long times. While all curves exhibited the same contraction behavior at short times (for all curves, we obtained $t_{0} = \tau_{1} = 410\, {\text{s}}$), distinct differences were detected at later stages. It is difficult to represent this additional relaxation by a single exponential function, especially for the non-monotonic curve #5. However, such non-monotonic behavior is not always observed and depends on parameter settings, in particular film thickness and preparation/annealing conditions. A similar dependence on parameter settings was also found for deformed polymer melts [47–50]. Of course, equilibrated samples should and do exhibit a monotonous behavior, often represented by an Arrhenius-behavior. A comparison of the behavior of annealed (almost equilibrated) and as prepared thin films has been presented in our previous paper [21]. Future experiments may help to identify the conditions of thermal treatment which allow that non-equilibrated polymers can generate a non-monotonic behavior, i.e., a transient period of re-elongation in the course of shrinkage.

### Temperature-sweep creep experiments

**Fig. 6:**
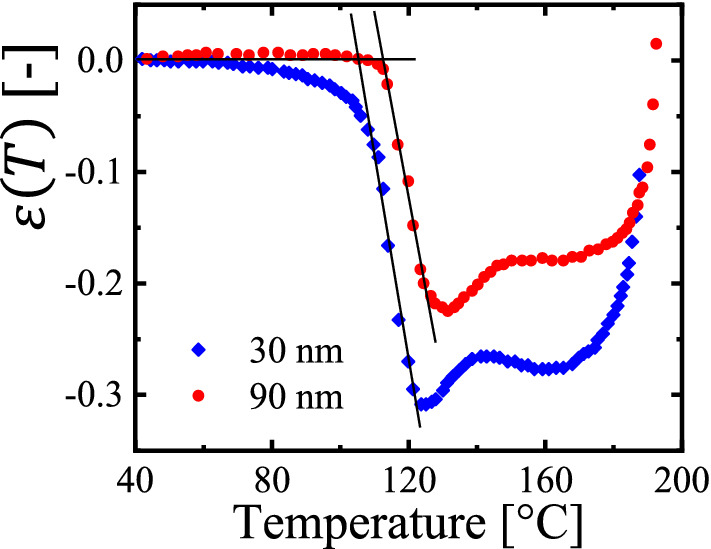
Indications for a thickness-dependent behavior. Strain $\varepsilon \left( T \right)$ as a function of temperature for filaments made from 30 and 90 nm thick polystyrene ($M_{{\text{W}}}$ = 524 kg/mol) films in a temperature sweep creep experiment performed at a heating rate of 4 °C/min with an applied load of 10 kPa and 5 kPa, respectively

When increasing the temperature beyond the glass transition temperature $T_{{\text{g}}}$, the elastic modulus of polymers drops considerably from its glassy to the rubbery value [7–9]. Thus, when performing creep experiments a in a temperature-sweep mode, we can determine the glass transition temperature. Accordingly, we executed creep experiments while increasing temperature for filaments of thin crumpled films prepared by spin coating. As derived from dewetting [22] and the here employed creep experiments [21], the magnitude of the residual stresses in spin coated thin polystyrene films is comparable to the rubbery modulus. Thus, we anticipated that filaments start to contract when their elastic modulus became comparable to the value of the residual stresses. Figure 6 shows the results of a creep experiment performed in a temperature sweep mode at a heating rate of about 4 °C/min for two filaments made from a 30 nm and a 90 nm thick as-cast polystyrene ($M_{\text{W}}$ = 524 kg/mol) film, respectively.

Within the resolution of our approach, the filament made from a 90 nm film showed no detectable contraction up to ca. 109 °C, a value comparable to $T_{{\text{g}}}$ of bulk polystyrene [51–54]. However, for the filament made from a 30 nm film, the onset of contraction was detectable beyond experimental uncertainty at a temperature of about 50 °C.

For melt-spun polymer fibers, similar sub-$T_{{\text{g}}}$ contraction has been observed widely, even at temperatures far below the nominal glass transition [13–17]. Here, we would like to suggest that the presence of preparation-induced residual stresses may represent another possibility for generating thickness-dependent changes in behavior, often attributed to changes in the value of $T_{{\text{g}}}$ [51–54]. As proposed in [13–17], the energy “stored” in non-equilibrated polymers (frozen-in entropic forces) can be invested for an activation of relaxation processes below $T_{{\text{g}}}$ by overcoming local potential barriers.

Interestingly, isothermal neutron scattering experiments on rapidly elongated polyisoprene melts [48] showed that the mean radius of gyration of the deformed polymers decreased monotonously in the stretching direction while a non-monotonic expansion behavior was observed in the orthogonal directions. The chains initially compressed orthogonal to the stretching direction became even more compressed and only started to expand at times longer than the Rouse time [48]. Despite these differences between these neutron scattering experiments and the here performed creep tests, it would be interesting to explore if and to what extent the relaxation of the radius of gyration of deformed polymer chains contributes to the observed non-monotonic behavior of $\varepsilon(\check{t})$.

## Conclusions

There are, of course, many experimental and theoretical approaches which help improve our understanding consequences of processing-induced non-equilibrium conformations of polymers. Our simple and macroscopic approach has the advantage of being highly sensitive to changes induced by preparation (processing, Fig. 4) and post-preparation relaxations (e.g., induced by annealing, Fig. 5). For example, the observed relaxations differed clearly for films spin coated under different conditions (see Fig. 4). Interestingly, relaxations were not always leading to monotonic shrinkage (see Fig. 5) and suggested a spectrum of distinguishable relaxation regimes with different timescales. Thus, our experiments can provide insight into how non-equilibrated polymers “forget”, how they lose their memory induced by processing.

The similar behavior of non-equilibrated polymer chains in drawn melt-spun fibers and spin coated thin films is not expected at first glance. One may anticipate that some of the various ways of how non-equilibrated polymers relax and impact polymer properties may be similar. It is, however, surprising that both the preparation-induced residual stresses in spin coated thin films and the thermal stresses in drawn melt-spun fibers yield similar results (see Fig. 3). Further systematic experiments are needed to corroborate these similarities or to highlight differences. Given the sensitivity of the here chosen creep experiments, we believe that more insight can be gained from exploring these differences systematically. Thus, we anticipate that future experiments [5, 11, 55] and theory/simulations [56–62] will identify general concepts for describing properties of non-equilibrated polymers and their relaxation behavior.
